# Global burden of emergency and operative conditions: an analysis of Global Burden of Disease data, 2011–2019

**DOI:** 10.2471/BLT.24.292412

**Published:** 2025-01-20

**Authors:** Sabrina Wimmer, Shreeja Sarabu, Emilie Calvello Hynes, Mary Louisa Plummer, Maeve Sophia Bognini, Meskerem Aleka Kebede, Martilord Ifeanyichi, Hassan Daoud, Mariam Dahir, Rachel Hargest, Rocco Friebel

**Affiliations:** a Global Surgery Policy Unit, LSE Health, London School of Economics and Political Science, Houghton Street, London WC2A 2AE, England.; b Clinical Services and Systems Unit, Department of Integrated Health Services, World Health Organization, Geneva, Switzerland.; c Suuban Center for Health System Strengthening, Hargeisa, Somalia.; d Royal College of Surgeons of England, London, England.

## Abstract

**Objective:**

To estimate the global burden of conditions requiring emergency or operative care and to investigate variations over time and between countries.

**Methods:**

We obtained data on deaths and disability-adjusted life years (DALYs) lost from the Global Burden of Disease database for 193 countries covering 2011 to 2019. We defined emergency conditions as conditions that, if not diagnosed and treated within hours to days of onset, often lead to serious physical or mental disability or death. We defined operative conditions as conditions that may require the expertise of a surgically trained provider and these conditions were identified using a modified Delphi consensus process.

**Findings:**

In 2019, emergency conditions accounted for 27 167 926 deaths and 1 015 000 000 DALYs globally, and operative conditions accounted for 17 648 680 deaths and 619 600 000 DALYs. Conditions classified as emergency-and-operative conditions accounted for 6 966 425 deaths and 303 344 808 DALYs. For emergency conditions, the per capita burden of deaths and DALYs was greatest for low-income countries. Between 2011 and 2019, deaths and DALYs due to emergency conditions decreased, whereas deaths due to operative conditions increased slightly. These trends may have been driven by strengthened prevention and early detection mechanisms, improved emergency care provision or epidemiological changes. However, because emergency and operative conditions were defined differently, it may not be valid to compare trends directly.

**Conclusion:**

The high global burden of emergency and operative conditions identified underscores the importance of strengthening and scaling up integrated emergency, critical and operative care internationally.

## Résumé

**Objectif:**

Estimer la charge mondiale des affections nécessitant des soins d’urgence ou des interventions chirurgicales et étudier les variations dans le temps et entre les pays.

**Méthodes:**

Nous avons obtenu des données sur les décès et les années de vie corrigées de l’incapacité (AVCI) perdues à partir de la base de données sur la charge mondiale de morbidité pour 193 pays, de 2011 à 2019. Nous avons défini les affections nécessitant des soins d’urgence comme des pathologies qui, si elles ne sont pas diagnostiquées et traitées dans les heures ou les jours qui suivent leur apparition, entraînent souvent une incapacité physique ou mentale grave, voire la mort. Nous avons défini les affections opératoires comme des états pouvant nécessiter l’expertise d’un prestataire ayant reçu une formation chirurgicale; ces états ont été identifiés à l’aide d’une méthode de Delphes modifiée de recherche de consensus.

**Résultats:**

En 2019, les états d’urgence ont été à l’origine de 27 167 926 décès et de 1,015 milliard d’AVCI dans le monde, et les états opératoires de 17 648 680 décès et de 619,6 millions d’AVCI. Les affections classées dans la catégorie des affections opératoires ou nécessitant des soins d’urgence ont été à l’origine de 6 966 425 décès et de 303 344 808 AVCI. Pour les affections nécessitant des soins d’urgence, la charge de morbidité par habitant en termes de décès et d’AVCI était la plus importante dans les pays à faible revenu. Entre 2011 et 2019, le nombre de décès et d’AVCI dus à des situations d’urgence a diminué, tandis que le nombre de décès dus à des affections opératoires a légèrement augmenté. Ces tendances peuvent s’expliquer par le renforcement des mécanismes de prévention et de détection précoce, l’amélioration de l’offre de soins d’urgence ou des changements épidémiologiques. Cependant, comme les affections nécessitant des soins d’urgence et les affections opératoires ont été définies différemment, la comparaison directe des tendances n’est peut-être pas valable.

**Conclusion:**

La lourde charge que représentent les affections nécessitant des soins d’urgence et les interventions chirurgicales dans le monde souligne l’importance de renforcer et d’étendre les soins d’urgence intégrés, les soins critiques et les interventions chirurgicales à l’échelle internationale.

## Resumen

**Objetivo:**

Estimar la carga mundial de afecciones que requieren atención urgente o quirúrgica e investigar las variaciones a lo largo del tiempo y entre países.

**Métodos:**

Se obtuvieron datos sobre muertes y años de vida ajustados por discapacidad (AVAD) perdidos de la base de datos Global Burden of Disease para 193 países que abarcan de 2011 a 2019. Se definieron como afecciones de emergencia las enfermedades que, si no se diagnostican y tratan en las horas o días siguientes a su aparición, suelen causar discapacidad física o mental grave o la muerte. Se definieron las afecciones de tipo quirúrgico como aquellas que pueden requerir la experiencia de un profesional con formación quirúrgica, y estas afecciones se identificaron mediante un proceso de consenso Delphi modificado.

**Resultados:**

En 2019, las afecciones de emergencia representaron 27 167 926 muertes y 1 015 000 000 AVAD a nivel mundial y las afecciones de tipo quirúrgico representaron 17 648 680 muertes y 619 600 000 AVAD. Las afecciones clasificadas como de emergencia y de tipo quirúrgico representaron 6 966 425 muertes y 303 344 808 AVAD. En el caso de las afecciones de emergencia, la carga per cápita de muertes y AVAD fue mayor en los países de ingresos bajos. Entre 2011 y 2019, las muertes y los AVAD debidos a afecciones de emergencia disminuyeron, mientras que las muertes debidas a afecciones de tipo quirúrgico aumentaron ligeramente. Estas tendencias pueden deberse al refuerzo de los mecanismos de prevención y detección precoz, a la mejora de la atención de urgencia o a cambios epidemiológicos. Sin embargo, dado que las afecciones de emergencia y de tipo quirúrgico se definieron de forma diferente, es posible que no sea válido comparar las tendencias de manera directa.

**Conclusión:**

La alta carga mundial de afecciones de emergencia y de tipo quirúrgico identificadas destaca la importancia de reforzar y aumentar la atención integrada de emergencia, crítica y quirúrgica a nivel internacional.

## ملخص

الغرض تقدير العبء العالمي للحالات التي تتطلب رعاية طارئة أو جراحية، والتحقيق في الاختلافات بمرور الوقت وبين الدول.

الطريقة حصلنا على بيانات عن الوفيات، ومعدلات سنوات العمر المعدلة بالإعاقة (DALY) المفقودة من قاعدة بيانات العبء العالمي للأمراض لـ 193 دولة تغطي الفترة من 2011 إلى 2019. لقد قمنا بتعريف الحالات الطارئة بأنها الحالات، التي إذا لم يتم تشخيصها وعلاجها في غضون ساعات إلى أيام من ظهورها، فغالبًا ما تؤدي إلى إعاقة بدنية أو عقلية خطيرة أو الوفاة. وقمنا بتعريف الحالات الجراحية بأنها الحالات التي قد تتطلب خبرة مقدم الخدمة المُدرّب جراحيًا، وتم تحديد هذه الحالات باستخدام عملية إجماع Delphi المعدلة.

النتائج في عام 2019، تسببت حالات الطوارئ في 27167926 حالة وفاة، و1015000000 من سنوات العمر المعدلة بالإعاقة على مستوى العالم، بينما تسببت الحالات الجراحية في 17648680 حالة وفاة، و619600000 من سنوات العمر المعدلة بالإعاقة. أما الحالات المصنفة على أنها حالات طارئة وجراحية، فقد تسببت في 6966425 حالة وفاة، و303344808 من سنوات العمر المعدلة بالإعاقة. وبالنسبة للحالات الطارئة، كان العبء الفردي من الوفيات وسنوات العمر المعدلة بالإعاقة، الأكبر في الدول ذات الدخل المنخفض. وبين عامي 2011 و2019، انخفضت الوفيات وسنوات العمر المعدلة بالإعاقة بسبب الحالات الطارئة، بينما زادت الوفيات بسبب الحالات الجراحية بشكل طفيف. وربما كانت هذه الاتجاهات مدفوعة بتعزيز آليات الوقاية والكشف المبكر، أو تحسين تقديم الرعاية الطارئة، أو التغيرات الوبائية. ومع ذلك، نظرًا لأن الحالات الطارئة والجراحية قد تم تعريفهما بشكل مختلف، فقد لا يكون من الصحيح مقارنة الاتجاهات بشكل مباشر.

الاستنتاج إن العبء العالمي المرتفع للحالات الطارئة والجراحية التي تم تحديدها، يؤكد على أهمية تعزيز وتوسيع نطاق الرعاية الطارئة والحرجة والجراحية المتكاملة على المستوى الدولي.

## 摘要

**目的:**

估计劝酒在需要接受急症护理或手术治疗的疾病方面的负担，并探究该负担在不同时期和不同国家之间的差异。

**方法:**

我们从全球疾病负担数据库获取了 193 个国家 2011 年至 2019 年期间的死亡和伤残调整生命年 (DALY) 损失数据。急症是指，如果在发病后数小时至数天内未得到诊断和治疗，通常会导致严重的生理或智力缺陷甚至死亡的疾病。需要接受手术治疗的疾病是指，可能需要训练有素的专业人员实施手术的疾病，这些疾病可以通过采用改良后的德尔菲法来确定。

**结果:**

2019 年，全球急症导致 27,167,926 例死亡和 1,015,000,000 个 DALY 损失，需要接受手术治疗的疾病导致 17,648,680 例死亡和 619,600,000 个 DALY 损失。被归类为急症且需要接受手术治疗的疾病导致 6,966,425 例死亡和 303,344,808 个 DALY 损失。就急症而言，低收入国家的人均死亡和伤残调整生命年损失负担最大。2011 年至 2019 年期间，因急症导致的 DALY 损失有所下降，而需要接受手术治疗的疾病导致的死亡人数则略有上升。这些趋势可能是由于加强了预防和早期检测机制、改善了紧急服务或流行病学的变化所促成的。然而，由于急症和需要接受手术治疗的疾病的定义不同，直接比较这些趋势可能并不妥当。

**结论:**

所确定的急症和需要接受手术治疗的疾病的全球负担沉重，这凸显了在国际上加强推行和扩大使用综合性急症护理、危重症监护和手术治疗的重要性。

## Резюме

**Цель:**

Оценить глобальное бремя состояний, требующих неотложной помощи или оперативного вмешательства, и изучить различия во времени и между странами.

**Методы:**

Из базы данных «Глобальное бремя болезней» были получены данные о смертности и годах жизни, скорректированных по нетрудоспособности (DALY), в 193 странах за период с 2011 по 2019 год. Под термином «состояния, требующие неотложной помощи» подразумеваются состояния, которые, если их не диагностировать и не начать лечить в течение нескольких часов или дней с момента возникновения, часто приводят к инвалидности вследствие соматических или психических нарушений или к смерти. Под термином «состояния, требующие оперативного вмешательства» подразумеваются состояния, для лечения которых может потребоваться опыт хирурга, и эти состояния были определены с помощью модифицированного процесса согласования в Delphi.

**Результаты:**

В 2019 году на долю состояний, требующих неотложной помощи, пришлось 27 167 926 смертей и 1 015 000 000 лет жизни, скорректированных по нетрудоспособности во всем мире, а на долю состояний, требующих оперативного вмешательства, – 17 648 680 смертей и 619 600 000 лет жизни, скорректированных по нетрудоспособности. На состояния, классифицированные как требующие одновременно и неотложной помощи, и оперативного вмешательства, пришлось 6 966 425 смертей и 303 344 808 лет жизни, скорректированных по нетрудоспособности. Что касается состояний, требующих неотложной помощи, бремя смертей и показатели DALY на душу населения были наиболее значительными в странах с низким уровнем дохода. В период с 2011 по 2019 год количество смертей и показатель DALY, обусловленные состояниями, требующими неотложной помощи, снизились, в то время как количество смертей, обусловленных состояниями, требующими оперативного вмешательства, несколько возросло. Эти тенденции могли быть обусловлены укреплением механизмов профилактики и раннего выявления, улучшением оказания неотложной помощи или эпидемиологическими изменениями. Однако, поскольку состояния, требующие неотложной помощи, и состояния, требующие оперативного вмешательства, определялись по-разному, прямое сравнение тенденций может быть неправомерным.

**Вывод:**

Высокое глобальное бремя состояний, требующих неотложной помощи, и состояний, требующих оперативного вмешательства, подчеркивает важность укрепления и расширения масштабов комплексной неотложной, критической и хирургической помощи на международном уровне.

## Introduction

In passing resolution 76.2 *Integrated emergency, critical and operative care for universal health coverage and protection from health emergencies* – the so-called ECO resolution – at the World Health Assembly in 2023,^1^ World Health Organization (WHO) Member States pledged to strengthen their health systems to provide high-quality, integrated, emergency, critical and operative care. Historically, health systems have often adopted a vertical approach that focused health services on specific population groups or conditions, such as maternal morbidity and mortality, particular infectious diseases or trauma. The ECO resolution emphasizes the importance of a horizontal alignment and the integration of health-care services along the patient pathway at all levels, from primary care to tertiary specialist care. Further, the coronavirus disease 2019 (COVID-19) pandemic highlighted the need for more resilient health systems that can better respond and adapt to external shocks and health emergencies. Stronger health systems are also necessary for attaining the United Nations sustainable development goals for 2030,^2^ particularly goal 3: to ensure healthy lives and promote well-being for all at all ages.^3^ This alignment and integration is especially important today because global progress towards achieving universal health coverage by 2030 has fallen behind, particularly for health service coverage.^4^

The World Health Assembly’s ECO resolution calls for greater political commitment to strengthening the planning and provision of integrated emergency, critical and operative care services, in anticipation of better population health. Policy-makers require sound evidence to develop national policies for the expansion of needs-based, integrated emergency, critical and operative care and to establish priorities for local settings. The objective of our study was to quantify the global, regional and national burden of conditions that may require emergency or operative care in terms of deaths and disability-adjusted life years (DALYs).

## Methods

### Definitions

There is no global consensus on definitions of emergency, critical or operative care or on the conditions that would require these types of care. Based on two previous studies using Global Burden of Disease data,^5^^,^^6^ we defined an emergency condition as, “a condition that, if not diagnosed and treated within hours to days of onset, often leads to serious physical or mental disability or death.” Although definitions and classifications of critical care have been proposed in recent studies,^7^^,^^8^ it is difficult to estimate the global critical care burden because the critical illness syndromes, such as sepsis and multiorgan dysfunction, associated with critical conditions are neither widely reported nor included in Global Burden of Disease data.^9^ Consequently, we excluded critical care from our study. However, critical care is provided for all conditions categorized as emergency conditions and for the majority of conditions categorized as operative conditions.^7^ We defined an operative condition as, “any condition that may require the expertise of a surgically trained provider,” and operative care as, “any measure that reduces the rates of physical disability or premature death associated with a surgical condition.”^10^ Although the Disease Control Priorities project applied a narrower definition and included only invasive procedures in its data analysis,^11^ it was acknowledged that surgical conditions can be managed using either a surgical procedure or a conservative approach. For example, an abscess can be incised and drained or it can be treated with antibiotics, and a splenic injury can be managed by emergency spleen removal or by monitoring, as is common in children.

### Data source and categories

Our study involved annual data from the publicly accessible Global Burden of Disease database for the years 2011 to 2019, before COVID-19 had a confounding influence on health-care delivery. We expressed the global, regional and national burden of emergency and operative conditions in terms of deaths and DALYs per 100 000 population using official country population estimates.^12^ A checklist for the Guidelines for Accurate and Transparent Health Estimates Reporting is available in the online repository.^13^^,^^14^

Global Burden of Disease data are classified using four levels. As an example: (i) level 1: noncommunicable diseases; (ii) level 2: cardiovascular diseases; (iii) level 3: stroke; and (iv) level 4: ischaemic stroke. We used the most detailed classification available for each condition listed. Chang et al. employed a Delphi consensus process to classify conditions according to their need for emergency care: (i) conditions that, if not addressed within hours to days of onset, commonly lead to serious disability or death; (ii) conditions commonly associated with acute decompensation that lead to serious disability or death; and (iii) non-emergency conditions.^6^ Others later adapted these categories and, to reduce the risk of overestimating emergency conditions, included only conditions in Chang et al.’s first category in their analyses.^5^ We used the adapted classification to identify emergency conditions. Remaining conditions were classified as non-emergency conditions (e.g. osteoarthritis and dementia).

For operative conditions, we performed a modified Delphi consensus process to classify all conditions listed in the Global Burden of Disease database as either operative or non-operative. The consensus process adhered to best practices, involving 12 participants across two rounds.^15^ We chose this number based on evidence of diminishing returns beyond 12. Participants, identified through networks such as the Royal College of Surgeons of England and WHO, were selected for their clinical expertise and geographic diversity. All had backgrounds in surgical specialties, anaesthesia, intensive care, emergency medicine or dentistry. They were based in seven low- and middle-income countries (Belize, Ethiopia, India, Malawi, Somalia, Sudan, and the United Republic of Tanzania) and four high-income countries (Sweden, Switzerland, the United Kingdom of Great Britain and Northern Ireland, and the United States of America). Participation was confidential, with identities kept anonymous during and after the Delphi process. Participants provided informed consent, understanding that involvement was voluntary and could be discontinued at any time. Additional information is provided in the online repository.^16^ We applied a consensus threshold of 67%. We did not categorize conditions listed in the Global Burden of Disease database that were too nonspecific to classify (e.g. other neonatal disorders and other malignant neoplasms) as either operative or non-operative. For nine conditions, no consensus could be reached: these included animal contact, sexual violence, rheumatic and nonrheumatic valvular heart disease, liver cancer and periodontal disease. We performed a literature search for each of these nine conditions and classified them as operative conditions if the treatment options described fell under our definition of operative care. 

A condition that was identified as both an emergency condition and an operative condition was classified as an emergency-and-operative condition. This third category differed from our broader group of emergency and/or operative conditions combined, which included all conditions that were either emergency conditions only, operative conditions only or emergency-and-operative conditions.

### Data analysis

In our baseline analysis, we present summary statistics on deaths and DALYs associated with emergency, operative and emergency-and-operative conditions in 2019. Temporal trends in all-age deaths and DALYs across 9 years (i.e. 2011 to 2019) and for 193 countries were assessed using panel data models. The analytical models included dummy variables for the Global Burden of Disease geographical regions and country income categories derived from 2022 World Bank classifications.^17^ Country-level fixed effects were used to account for unobserved variations in country characteristics that were assumed to be constant throughout the study period. As it was possible that the correlation between errors in observations in an individual country were greater than the correlation in errors between countries, our model used standard errors clustered at the country level. We repeated our analysis for deaths and DALYs linked to emergency conditions, operative conditions and emergency-and-operative conditions, respectively. A *P*-value less than 0.05 was considered significant. All analyses were conducted using Stata v. 16 SE (StataCorp LLC, College Station, United States of America).

## Results

We identified 193 countries for which information on deaths and DALYs linked to 272 conditions was available from the Global Burden of Disease database. Of the 272 conditions, we categorized 61 as emergency conditions and 211 as non-emergency conditions. In addition, 88 were categorized as operative conditions and 184 as non-operative conditions. Finally, 31 were categorized as emergency-and-operative conditions, and 118 were categorized as emergency and/or operative conditions. An overview of all classifications is presented in an online repository.^18^

**Fig. 1:**
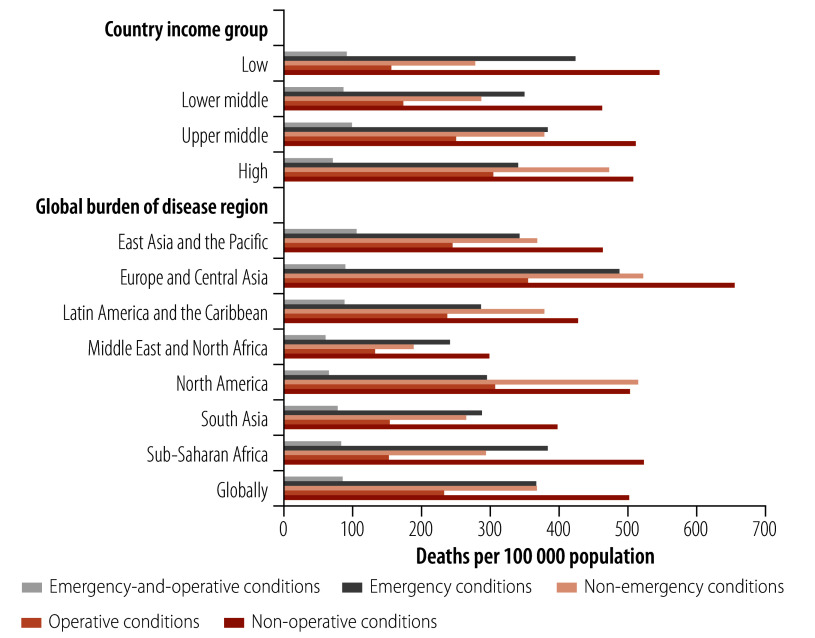
Mortality burden of emergency and operative conditions, by country income group and regional grouping, 2019

Note: We derived country income categories from 2022 World Bank classifications.^17^

**Fig. 2:**
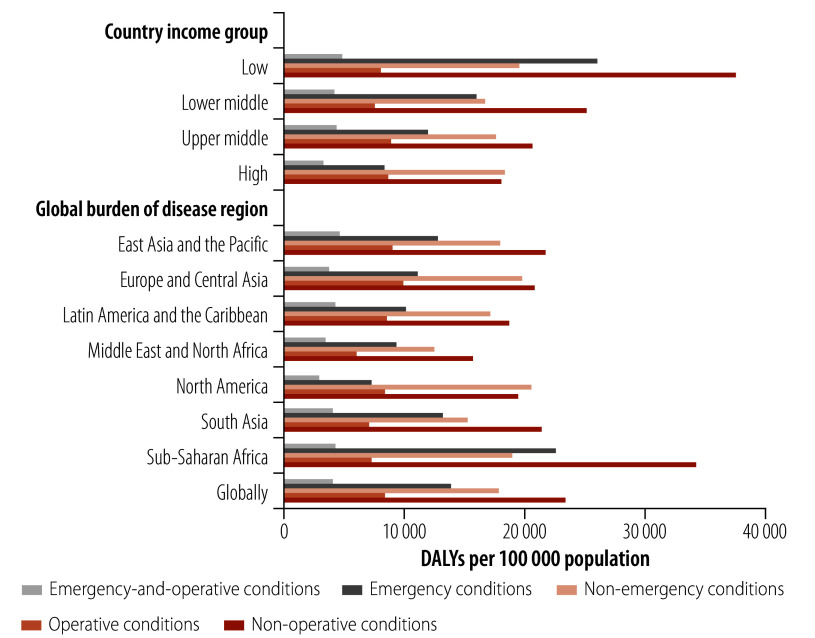
Disability burden of emergency and operative conditions, by country income group and regional grouping, 2019

In 2019, emergency and/or operative conditions accounted for 37 850 181 deaths (514.09 deaths per 100 000 population) and 1 331 300 000 DALYs (18 113.00 DALYs per 100 000 population) worldwide; and emergency-and-operative conditions accounted for 6 966 425 deaths (86.92 deaths per 100 000 population) and 303 344 808 DALYs (4070.95 DALYs per 100 000 population; Table 1; Fig. 1 and Fig. 2). Low-income countries reported the highest burden of DALYs associated with emergency-and-operative conditions (4894.41 DALYs per 100 000 population) and this burden decreased with the rise in income classification, such that the burden for high-income countries was 3316.45 DALYS per 100 000 population (Table 1 and Fig. 2). In contrast, the largest burden of deaths associated with emergency-and-operative conditions was reported for upper-middle-income countries (99.20 deaths per 100 000 population; Table 1 and Fig. 1). Regionally, the East Asia and the Pacific region reported the highest DALY burden associated with emergency-and-operative conditions (4626.38 DALYs per 100 000 population); followed by sub-Saharan Africa (4268.36 DALYs per 100 000 population); and Latin America and the Caribbean (4260.94 DALYs per 100 000 population; Table 1 and Fig. 2). The East Asia and the Pacific region also report the largest burden of deaths associated with emergency-and-operative conditions (107.14 deaths per 100 000 population; Table 1 and Fig. 1).

**Table 1:** Health burden of emergency and operative conditions, by country income group and regional grouping, 2019

Country characteristic^a^	Health burden													
	Emergency-and-operative conditions^b^ (*n* = 31)			Emergency conditions (*n* = 61)			Non-emergency conditions (*n* = 211)			Operative conditions (*n* = 88)			Non-operative conditions (*n* = 184)	
	Deaths per 100 000 population	DALYs per 100 000 population		Deaths per 100 000 population	DALYs per 100 000 population		Deaths per 100 000 population	DALYs per 100 000 population		Deaths per 100 000 population	DALYs per 100 000 population		Deaths per 100 000 population	DALYs per 100 000 population
**Country income group^c^**														
Low	92.08	4894.41		424.67	26 002.56		279.34	19 563.69		156.90	8045.56		547.11	37 520.61
Lower middle	87.60	4179.34		350.32	15 960.57		288.08	16 723.96		174.60	7513.32		463.81	25 171.16
Upper middle	99.20	4390.43		384.58	11 932.70		378.88	17 523.01		251.04	8855.13		512.43	20 600.53
High	72.78	3316.45		341.36	8 391.27		473.28	18 299.45		306.18	8661.58		508.46	18 029.14
**Global Burden of Disease region**														
East Asia and the Pacific	107.14	4626.38		343.23	12 749.70		369.26	17 922.99		245.72	8959.06		466.77	21 713.60
Europe and Central Asia	89.84	3726.49		488.87	11 087.61		522.98	19 726.37		355.43	9965.97		656.42	20 848.00
Latin America and the Caribbean	89.12	4260.94		287.29	10 113.45		379.75	17 134.33		237.73	8541.23		429.30	18 706.56
Middle East and North Africa	60.74	3491.08		242.09	9 262.74		189.63	12 503.19		132.83	6018.05		298.90	15 747.83
North America	66.39	2975.87		295.49	7 321.92		515.79	20 544.33		307.13	8391.94		504.14	19 474.33
South Asia	78.65	4041.05		287.97	13 166.99		265.56	15 272.37		154.64	7057.96		398.89	21 381.15
Sub-Saharan Africa	83.86	4268.36		383.85	22 562.76		294.09	18 955.84		153.47	7282.05		524.47	34 236.50
**Globally**	86.92	4070.95		367.18	13 872.48		368.93	17 811.71		233.83	8311.47		502.28	23 372.68

DALY: disability-adjusted life year.

^a^ The study included data from 193 countries.

^b^ An emergency-and-operative condition was defined as a condition that was classified as both an emergency and an operative condition.

^c^ Country income categories were derived from 2022 World Bank classifications.^17^

DALY: disability-adjusted life year.

Note: We derived country income categories from 2022 World Bank classifications.^17^

Emergency conditions were responsible for a substantial share of deaths and DALYs globally; namely, 27 167 926 deaths (367.18 deaths per 100 000 population) and 1 015 000 000 DALYs (13 872 DALYs per 100 000 population; Table 1; Fig. 1 and Fig. 2). Moreover, the per capita burden of emergency conditions was greatest in low-income countries (424.67 deaths and 26 002.56 DALYs per 100 000 population) and the burden generally decreased with the rise in income classification (Fig. 1 and Fig. 2). Considerable regional variations in DALYs due to emergency conditions were observed. For example, in sub-Saharan Africa, the reported figure was 22 562.76 DALYs per 100 000 population, which was more than three times that reported for the North America region (7321.92 DALYs per 100 000 population; Fig. 2).

In comparison, operative conditions accounted for 17 648 680 deaths (233.83 deaths per 100 000 population) and 619 600 000 DALYs (8311.47 DALYs per 100 000 population) globally (Table 1; Fig. 1 and Fig. 2). The burden of deaths linked to operative conditions was highest in high-income countries (306.18 deaths per 100 000 population) and lowest in low-income countries (156.90 deaths per 100 000 population; Table 1 and Fig. 1). The burden of DALYs linked to operative conditions was similar across all country income groups, with the highest burden being recorded for upper-middle-income countries (8855.13 DALYs per 100 000 population; Table 1 and Fig. 2). Regionally, the highest burden of deaths linked to operative conditions was reported for the Europe and Central Asia region (i.e. 355.43 deaths per 100 000 population) and the North America region (i.e. 307.13 deaths per 100 000 population; Fig. 1). Figures for the burden of deaths and DALYs in individual countries are presented in the online repository.^19^

Time trends in deaths and DALYs associated with emergency, operative and emergency-and-operative conditions derived using our panel data models are presented in Table 2 and Table 3, respectively, along with comparisons between country income groups and regional groupings. Overall, we found that the burden of deaths and DALYs decreased globally over time for emergency-and-operative conditions. There were substantial decreases in deaths and DALYs for emergency conditions, but a small increase in deaths and a decrease in DALYs for operative conditions. The decreases seen for emergency-and-operative conditions were driven by changes in deaths and DALYs for emergency conditions. Between 2011 and 2019, the per capita burden of deaths and DALYs linked to emergency-and-operative conditions was least in high-income countries (Table 2 and Table 3). The same was true for deaths and DALYs linked to emergency conditions: high-income countries reported the lowest per capita burden, and most associated deaths and DALYs occurred in low-income countries. Compared with high-income countries, lower-middle-income countries reported a significantly lower burden of deaths linked to operative conditions throughout the time period, whereas low-income countries reported a small but significantly higher burden of DALYs.

**Table 2:** Differences in mortality burden of emergency and operative conditions from 2011 to 2019 globally and by country income group and regional grouping

Variable	Difference in mortality^a^							
	Emergency-and-operative conditions^b^ (*n* = 31)			Emergency conditions (*n* = 61)			Operative conditions (*n* = 88)	
	Deaths per 100 000 population	*P*		Deaths per 100 000 population	*P*		Deaths per 100 000 population	*P*
**Year **								
2011	Reference	NA		Reference	NA		Reference	NA
2012	−0.16	NS		−5.75	< 0.05		0.76	NS
2013	−0.70	NS		−11.02	< 0.001		0.92	NS
2014	0.51	NS		−12.61	< 0.001		3.09	< 0.05
2015	0.05	NS		−14.86	< 0.001		4.27	< 0.001
2016	−1.49	NS		−19.37	< 0.001		3.60	< 0.05
2017	−2.00	NS		−21.82	< 0.001		4.22	< 0.001
2018	−3.06	< 0.05		−23.61	< 0.001		4.92	< 0.001
2019	−4.49	< 0.001		−25.51	< 0.001		5.16	< 0.001
**Country income group^c,d^**								
High	Reference	NA		Reference	NA		Reference	NA
Low	53.67	< 0.001		246.90	< 0.001		−16.33	NS
Lower middle	31.12	< 0.001		145.60	< 0.001		−38.30	< 0.05
Upper middle	32.19	< 0.001		106.20	< 0.001		−21.31	NS
**Global Burden of Disease region^c^**								
Europe and Central Asia	Reference	NA		Reference	NA		Reference	NA
East Asia and the Pacific	3.45	NS		−201.80	< 0.001		−96.14	< 0.001
Latin America and the Caribbean	−12.33	NS		−239.30	< 0.001		−111.70	< 0.001
Middle East and North Africa	−26.96	< 0.001		−280.10	< 0.001		−194.20	< 0.001
North America	−14.04	NS		−159.50	NS		−66.10	NS
South Asia	−34.51	< 0.05		−294.80	< 0.001		−172.60	< 0.001
Sub-Saharan Africa	−31.62	< 0.001		−192.10	< 0.001		−174.80	< 0.001

NA: not applicable; NS: not significant.

^a^ We determined differences in mortality between years, country income groups and regional groupings using panel data models covering 193 countries and 1737 observations for each category of condition.

^b^ We defined an emergency-and-operative condition as a condition that was classified as both an emergency and an operative condition.

^c^ We derived differences in mortality between country income groups and regional groupings using data for 2011–2019.

^d^ We derived country income categories from 2022 World Bank classifications.^17^

**Table 3:** Differences in disability burden of emergency and operative conditions from 2011 to 2019 globally and by country income group and regional grouping

Variable	Difference in disability^a^							
	Emergency-and-operative conditions^b^ (*n* = 31)			Emergency conditions (*n* = 61)			Operative conditions (*n* = 88)	
	DALYs per 100 000 population	*P*		DALYs per 100 000 population	*P*		DALYs per 100 000 population	*P*
**Year**								
2011	Reference	NA		Reference	NA		Reference	NA
2012	−4.39	NS		−446.50	< 0.01		−9.65	NS
2013	−41.35	NS		−854.00	< 0.01		−54.94	NS
2014	−6.75	NS		−1 127.00	< 0.01		−19.86	NS
2015	−74.28	NS		−1 517.00	< 0.01		−72.94	NS
2016	−129.80	< 0.1		−1 883.00	< 0.01		−131.90	< 0.1
2017	−185.20	< 0.01		−2 238.00	< 0.01		−187.00	< 0.01
2018	−277.90	< 0.01		−2 607.00	< 0.01		−265.30	< 0.01
2019	−372.70	< 0.01		−2 913.00	< 0.01		−345.30	< 0.01
**Country income group^c,d^**								
High	Reference	NA		Reference	NA		Reference	NA
Low	2931.00	< 0.01		17 320.00	< 0.01		2864.00	< 0.01
Lower middle	1314.00	< 0.01		8 315.00	< 0.01		871.30	< 0.1
Upper middle	1242.00	< 0.01		3 703.00	< 0.01		852.70	< 0.1
**Global Burden of Disease region^c^**								
Europe and Central Asia	Reference	NA		Reference	NA		Reference	NA
East Asia and the Pacific	353.00	NS		−1 616.00	NS		−1444.00	< 0.01
Latin America and the Caribbean	169.90	NS		−2 426.00	< 0.1		−1764.00	< 0.01
Middle East and North Africa	6.52	NS		−4 605.00	< 0.01		−3550.00	< 0.01
North America	−406.40	NS		−2 553.00	NS		−1560.00	NS
South Asia	−678.80	NS		−3 873.00	NS		−3578.00	< 0.01
Sub-Saharan Africa	−785.60	< 0.1		5 070.00	< 0.01		−3749.00	< 0.01

DALY: disability-adjusted life year; NA: not applicable; NS: not significant.

^a^ We determined differences in disability between years, country income groups and regional groupings using panel data models covering 193 countries and 1737 observations for each category of condition.

^b^ We defined an emergency-and-operative condition as a condition that was classified as both an emergency and an operative condition.

^c^ We derived differences in disability between country income groups and regional groupings using data for 2011–2019.

^d^ We derived country income categories from 2022 World Bank classifications.^17^

## Discussion

We estimated the burden of emergency and/or operative conditions globally to be 37 850 181 deaths and 1 331 300 000 DALYs in 2019 alone. This high level underscores the critical importance of strengthening and scaling up integrated emergency, critical and operative care, as emphasized in the 2023 World Health Assembly’s ECO resolution.^1^

Previous research using regional data for 1990 to 2015 from the Global Burden of Disease database found that an estimated 51% of deaths and 42% of DALYs globally were due to emergency conditions, and that the number of deaths and DALYs was inversely correlated with the World Bank’s country income classification.^5^ Injury, ischaemic heart disease, lower respiratory tract infection and haemorrhagic stroke accounted for the majority of emergency conditions in high- and upper-middle-income countries: 84% and 79% of all emergency conditions in these country groups, respectively. However, these conditions were also responsible for a substantial burden in lower-middle- and low-income countries: 39% and 49% of years of life lost in these country groups, respectively. Another study, which used 2010 Global Burden of Disease data and applied a broader definition of emergency conditions,^6^ estimated that emergency conditions accounted for 90% of deaths and 84% of DALYs globally. The highest burden was observed in low-income countries, where reported emergency care utilization rates were consistently lower than in other countries.^6^^,^^20^ We found that, although the estimated burden (both deaths and DALYs) of emergency conditions decreased across the study period globally, these conditions remained a substantial cause of death and disability. We also found that deaths linked to operative conditions increased slightly during the study period, whereas DALYs due to operative conditions decreased significantly from 2017 onwards. These trends may have been driven by strengthened prevention and early detection mechanisms, improved emergency care provision or epidemiological changes (e.g. an increase in the burden of noncommunicable diseases). However, because of differences in the way emergency and operative conditions were defined, it may not be valid to compare trends in their burdens directly.

The balance between the surgical and conservative management of operative conditions will likely vary according to the availability of resources, clinical presentation and surgical subspecialty. Previous research in the United States found that surgical procedures were performed for conditions in every 2010 Global Burden of Disease subcategory, with the highest surgical frequencies for musculoskeletal conditions (84.0%) and neoplasms (61.4%).^11^ In low- and middle-income countries, over 60% of surgical procedures were performed for emergency conditions in 2015.^11^ The third edition of *Essential surgery: disease control priorities* reported in 2015 that operative conditions were associated with 4.7 million deaths and 340 million DALYs each year, but acknowledged that these figures do not capture common operative conditions such as bowel obstruction or gallbladder disease.^11^ Our findings confirm the high burden of operative conditions, both generally and in high-income settings. Given the disruption in operative service delivery that occurred during the COVID-19 pandemic after our study period, it is likely that the burden of operative conditions would have increased further, with important implications for population health.^21^

Our classification of emergency-and-operative conditions highlighted conditions for which there was a particular need for a rapid, coordinated and multidisciplinary care. One such one condition is maternal haemorrhage, a major cause of maternal mortality, which accounted for 46 429 deaths and 3 085 190 DALYs globally in 2019. Both immediate resuscitation measures and potential surgical interventions, such as uterine artery ligation or hysterectomy, are required to save lives. Similarly, appendicitis is an emergency-and-operative condition. This condition resulted in 33 341 deaths and 1 498 796 DALYs globally in 2019. Treatment often requires early recognition and prompt surgical attention to prevent complications, such as perforation and peritonitis.

Emergency, critical and operative conditions create an immense economic burden. Between 2015 and 2030, operative conditions alone were estimated to result in 12.3 trillion United States dollars (US$) in lost economic productivity.^22^ Considerable public and private investment is required to strengthen the planning and provision of emergency, critical and operative care services needed to meet the health needs of the population, improve health system resilience and ensure a secure public health system.^23^ For example, in 2023 the cost of scaling up operative care in low- and middle-income countries was estimated to be US$ 300 billion.^24^ However, no country that has developed a national surgical obstetrics and anaesthesia plan has committed the necessary funding.^25^ Moreover, although health expenditure has been growing faster than the economy across many low- and middle-income countries,^26^^,^^27^ there remain major barriers to accessing emergency, critical and operative care, such as the need for high out-of-pocket payments.^24^^,^^28^ These barriers probably account for some of our findings, particularly in low-income settings.

The World Health Assembly’s ECO resolution calls for the standardization and disaggregation of data collection to: (i) accurately characterize and report disease burdens and, thereby, identify high-yielding mechanisms for improving the coordination, safety and quality of delivery of emergency, critical and operative care; and (ii) demonstrate how integrated care can contribute to meeting national targets, achieving health programme goals and attaining the sustainable development goals. Towards that end, there is a need for a comprehensive global measurement framework for, and indicators of, disease burden to improve the data available and support the research needed to guide evidence-based policy development and priority setting at the local level.^24^^,^^29^ Our study provides a transparent approach to classifying emergency and operative conditions that was embedded within a global consensus exercise. This approach enabled us to obtain detailed country-level estimates of the burden of these conditions that can be used to inform policy development and investment decisions at national and international levels.^30^^,^^31^

Our study has limitations. First, there is no global consensus on the definition of emergency, critical or operative conditions. Based on a previous study,^5^ we used a narrow classification of emergency conditions. As a result, we excluded some urgent medical conditions, such as diabetes mellitus and human immunodeficiency virus/acquired immunodeficiency syndrome, that can lead to acute decompensation requiring emergency care if left untreated and that can result in serious morbidity or death. To categorize operative conditions, we conducted an international Delphi consensus exercise involving participants with a range of clinical backgrounds from low-, middle- and high-income countries. Although we aimed to include individuals covering a diverse range of backgrounds and settings, we acknowledge that their responses may have been influenced by the local availability of resources, local treatment guidelines and their personal practices. For a small number of operative conditions, no consensus could be reached and the literature was consulted after discussion with participants instead of undertaking further rounds of the Delphi process. Second, the different ways in which we defined emergency and operative conditions means that, although both definitions might be useful for obtaining broad estimates of the burden associated with a particular type of care, their validity for comparing the burdens of emergency and operative conditions directly was limited.

More broadly, our study was affected by limitations in Global Burden of Disease data themselves and by the extent to which specific conditions can be equated to specific types of care. Global Burden of Disease data may be limited by variations in data sources and quality, especially in data from countries where hospital records and death registration are relatively incomplete.^32^^,^^33^ Such variations could reduce the validity of comparisons across regions and country income groups. Further, although the type of condition can broadly be used to indicate the type of care needed, the extent to which a specific type of care is required for a specific condition may vary greatly. For example, a cyclist road injury is categorized as an operative condition but not all cyclist road injuries will require surgery. Nonetheless, our study builds on previous research and uses a uniform method and terminology, thereby enabling valid comparisons to be made across time, regions and country income groups. Future studies could include estimates of the likelihood that emergency or operative care would be needed for each condition, which would further refine mortality and disability estimates.

In addition, although our study reported the burden of emergency and/or operative conditions, we were not able to estimate how much of the burden could be avoided by strengthening emergency, critical and operative care. Other actions, such as investing in prevention, could also affect the avoidable and unavoidable burden of emergency and operative care and should be considered as part of a more holistic approach to improving population health. Finally, our study used Global Burden of Disease data from before 2020, when the COVID-19 pandemic began. The impact of the major shock to the provision of emergency, critical and operative care caused by the pandemic and the subsequent recovery of health-care systems will need to be assessed by future research.

In conclusion, the high global burden of emergency and operative conditions we found in our study underscores the importance of strengthening and scaling up integrated emergency, critical and operative care, as emphasized in the 2023 World Health Assembly’s ECO resolution. A substantial proportion of the world's leading causes of death and morbidity could be addressed through the provision of emergency and operative care. Consequently, a global commitment to improving the planning and provision of integrated emergency, critical and operative care has the potential to meet the health needs of the population, improve health system resilience and ensure a secure public health system. Towards that end, it is vital to: (i) create a shared vision for emergency, critical and operative care by developing a global strategy and action plan; (ii) support leadership on emergency, critical and operative care within national health ministries; (iii) enhance WHO’s emergency, critical and operative capacity at all levels; and (iv) monitor implementation of the ECO resolution.
